# How Important Are Genetic Diversity and Cultivar Uniformity in Wheat? The Case of Gliadins

**DOI:** 10.3390/genes15070927

**Published:** 2024-07-16

**Authors:** Eugene Metakovsky, Viktor A. Melnik, Laura Pascual, Colin W. Wrigley

**Affiliations:** 1 Department of Biotechnology-Plant Biology, School of Agricultural, Food and Biosystems Engineering, Universidad Politécnica de Madrid, 28040 Madrid, Spain; 2 Vavilov Institute of General Genetics, Russian Academy of Sciences, Moscow 119991, Russia; 3 Queensland Alliance for Agriculture and Food Innovation (QAAFI), University of Queensland, Brisbane, QLD 4072, Australia

**Keywords:** wheat, biotypes, DUS, genetic erosion

## Abstract

Improvements in self-pollinated crops rely on crosses between different genotypes. It has been suggested that the repeated use of “the best” genotypes may lead to the restriction of the genetic diversity of the crop. In wheat, the analysis of gliadin (storage protein) polymorphism has provided evidence that genetic diversity was high and stable throughout the 20th century. Moreover, a worldwide analysis of gliadin polymorphism shows that genetic diversity is structured spatially across countries and their regions. Therefore, the analysis of gliadin genotypes in a given grain sample can provide reliable information about the origin of grains in this sample. An unexpected finding is that many registered common wheat cultivars are genetically non-uniform and composed of authentic biotypes (genotypically related lines originated from the initial cross) in spite of current crop-registration rules that include a strict demand for each new cultivar to be genetically uniform (DUS rules). In summary, the results suggest that each cultivar is the fruit of joint effects of a breeder and of a region’s environmental factors. We believe this finding will not be restricted to wheat and suggest there may be a need to re-evaluate relevant rules of cultivar registration for crop species in general.

## 1. History: Genetic Diversity Needed

There was a time when “wheat was wheat”, and there was little concern about segregating the harvested grain according to quality type. Even the homogeneity of the sown seed was uncertain.

**Figure 1:**
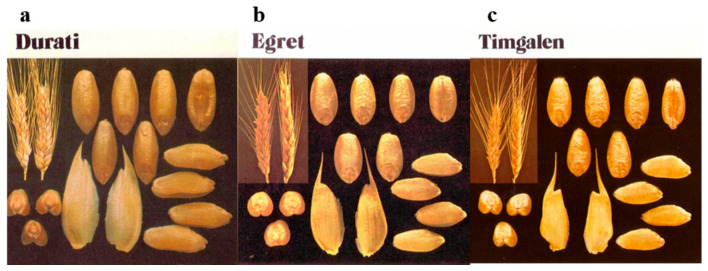
Spikes and grains of Australian wheat varieties: (**a**) the cultivar “Durati” (*T. durum*) [2]; (**b**) the cultivar “Egret”, (*T. aestivum*) [3]; and (**c**) the cultivar “Timgalen” (*T. aestivum*) [3]. For comparison, the three figures of cultivars are shown at the same scale.

A likely exception appeared when the distinction was made between the two major species of wheat, “common wheat” (*Triticum aestivum*, genetically hexaploid), with current annual production worldwide of about eight hundred million tonnes, and “durum wheat”, a distinct species suited for pasta production (*T. durum*, tetraploid), with annual production worldwide of some forty million tonnes. Durum wheat is clearly distinguished from common wheat by spike and grain characteristics (Figure 1a,b). Hence, the “wheat is wheat” concept has long been separated into two types of “wheat” (distinct species) in accordance with their different end-use destinations. Nevertheless, even in recent wheat examples, some grain samples of hexaploid wheat may still carry unrecognized admixtures of tetraploids, and vice versa [1].

Spike and grain attributes do not reliably distinguish between the quality types of many common wheat varieties (Figure 1b,c), such as animal feed versus milling to flour for the production of bread, cakes, noodles, and for gluten–starch manufacture and motor fuel production. A century or so ago, flour millers had to accept unsegregated common wheat grain as a commodity to serve the specific needs of the various flour users. Recent studies of old varieties and local landraces of common wheat worldwide have shown high levels of intra-varietal non-uniformity and genetic diversity [4,5,6,7].

With little concern in the industry for quality differences among common wheat varieties, there came pressure from flour millers to encourage early wheat breeders to produce varieties specifically for better flour yield and dough properties suited to bread production versus general use. Much of that pressure for improved wheat quality was exerted all the way from England to Australia, because the British saw the far-away colony as a “bread bowl” for “the Mother Country”, facilitated by the fast clipper ships taking the quick route to Europe, via “the Horn” of South America.

The challenge to breed better-quality wheat was taken up by William Farrer at his farm in southern New South Wales (NSW, an eastern state of Australia). His travels around NSW in the 1880s as a surveyor had shown him that the wheat varieties from northern climates being grown at that time were inappropriate and quite unsuited to the new continent. So, Farrer (although trained as a mathematician) took to breeding, based on correspondence with scientists overseas.

To ensure that his new varieties would be accepted by British millers, Farrer enlisted the help of chemist Frederick Guthrie, in Sydney, to develop testing systems suited to his small grain samples in selecting good milling and baking lines from Farrer’s crossbred wheats [8]. From these many wheat lines came one named “Jonathan”, combining the adaptation of wheat from India (with a similar climate to NSW) with the baking quality of the Canadian “Fife” wheat, leading in turn to such new varieties being segregated for export as “N.S.W. Strong White” wheat [8,9]. Similar breeding and selection efforts in Canada at the time were also leading to the commercial segregation of better-quality varieties such as “Marquis”, also based on the “Fife” background [10]. Equivalent efforts were being directed towards grain quality for the wide range of agricultural crops, including both monocot and dicot species.

More recently, wheat breeders, growers and grain handlers have recognized the compositional requirements for the many uses of wheat. There have been changes in the breeding, growing and segregating of grain to tailor wheat composition to meet these needs, which include milling yield, protein content and quality, starch type and grain hardness.

Various combinations of these quality attributes are needed to suit the processing quality required for contrasting food and non-food products, such as leavened bread, flat (Arabic) bread, noodles, cakes, biscuits (cookies), pasta, animal feeds and industrial uses, including starch–gluten manufacture and motor fuel bioethanol. Accordingly, breeders and agronomists still strive to develop varieties (“cultivars”) designed to be internally uniform and suited to the specifications for each of the many wheat grades, both within wheat-growing countries and in international trade. Similar considerations now apply to cultivars for a wider range of agricultural crops.

The history of wheat improvement is a story of progressive technological advances, from the manual selection of stronger plants for future propagation, often through the individual efforts of farmers who, in turn, sold on their improved seed. Subsequent technological improvements include progressions through cross-pollination of plants that together are expected to produce improved progeny, involving commercial operations and more sophisticated breeding technologies, together with the need for the registration of new varieties and mechanisms to provide intellectual property protection for the breeders [11].

Plant breeders’ rights (PBR) have been established for the range of agricultural and horticultural crops. Most countries have adopted PBR regulations based on the principles of the International Union for the Protection of New Varieties of Plants (UPOV, from the French acronym). Cultivar registration involves establishing the Distinctness (D), Uniformity (U) and Stability (S) (DUS) of a new cultivar offered for registration. DUS requirements have initially been based on morphological characteristics. However, “science does not stand still”; thus, testing mechanisms have developed well beyond plant morphology to include biochemical and molecular technologies. In recent decades, laboratory methods have been devised to provide unequivocal identification of the variety, as is now also required for the allocation of breeder royalties associated with specific varieties.

## 2. The Problem

Gone now is the concept that “wheat is wheat”, with little concern for which variety is sown and delivered. The Green Revolution was one of the factors accenting the distinction among genotypes; it became clear that breeding and genetics had accentuated the advantages offered by specific newly bred varieties. No longer could growers ignore the need to focus on specific varieties—not only on those with yield advantages but also on varieties of superior wheat quality—those that would attract premium prices at market.

However, in the second half of the 20th century, it was realized that the successes of breeders and scientists in obtaining the best genotypes of a cultivated plant for particular end-use purposes may lead to a dangerous narrowing of the genetic diversity of its germplasm as a whole [12]. Indeed, the frequent use, in breeding programs, of a limited number of “the best” genotypes (from which have already originated, with higher probability, other registered cultivars of desirable quality) may reduce the genetic background of the crop’s germplasm, causing its erosion. The reduction in the genetic diversity of the germplasm of the cultivated crop may seriously compromise its further improvement [13,14,15].

## 3. Gliadin Polymorphism Provides the Solution

To answer to this new challenge in wheat breeding, an approach was needed to perform studies on the genetic diversity of cultivated plants and their possible change over time. Genetic markers were needed that could distinguish more genotypes than morphological characters.

Historically, the genetic markers mostly used for the description and differentiation of wheat genotypes are grain storage proteins, especially gliadins.

The three main types of gliadin polypeptides (so-called α-, γ- and ω-gliadins) differ greatly from each other in many characteristics including their amino acid composition and domain structure [16]. The one-dimensional EP in an acid buffer, pH < 3.2 (APAGE), reveals about 20–25 gliadins (EP bands) present in one grain of any homozygous common wheat genotype [17,18]. Moreover, it was firmly established that the set of gliadins is specific for a given wheat genotype and does not depend on the eco-climatic conditions of growing, the level of fertilizer applied, the grain protein content or incomplete germination [19,20,21]. So, the APAGE analysis of gliadin was considered optimal and adopted by the International Association for Cereal Science and Technology as a standard technique for the analysis of gliadin protein polymorphism [22].

There are six major and several minor gliadin-coding (*Gli*) loci in common wheat, with each of them showing multiple allelism. Any allelic variant of the *Gli* locus has a complex structure [16,23], encoding two or more certain electrophoretic bands differing in their electrophoretic mobility and staining intensity. The group of jointly inherited gliadin electrophoretic bands was called the “block” [24]. Allelic variants of one *Gli* locus differ in the number and electrophoretic mobility of gliadin bands composing the block [24,25]. Currently, more than 180 alleles, in sum, at the six major *Gli* loci were identified [25]. At the same time, the γ-gliadin-specific DNA markers could distinguish (using the PCR technique) only two alternative (“allelic”) variants at each *Gli-1* locus [26]. In fact, classical DNA-based markers could not identify more alleles at the *Gli-B1* locus in the set of worldwide common wheat cultivars than the APAGE [27]. Thus, due to their particularities, especially due to their enormous intraspecific polymorphism and described multiple allelism of their encoding genes, gliadins offer an excellent opportunity for identifying and distinguishing between wheat genotypes, and therefore for any genetic studies.

In order to answer the question if there is indeed some decrease in the wheat genetic diversity over time, a systematic description of the worldwide wheat germplasm using gliadin markers was performed. The grain samples for this analysis were obtained from genetic and breeding centers and laboratories from the countries of origin of cultivars and genotyped by the APAGE.

The results show that the genetic diversity of the wheat germplasm studied was very high and stable throughout the 20th century for each of the six major *Gli* loci. Only a slight decrease in genetic diversity for the *Gli-B1* locus may be noted in the last of the groups studied (Table 1). There has never been a single case of shared identity of gliadin genotype among more than 1000 unrelated common wheat cultivars worldwide studied [25].

Finally, the use of different genetic markers showed that there was no noticeable loss of polymorphism in different crops including wheat during the 20th century [28,29], although many alleles present in landraces and local varieties were not found in common wheat cultivars registered in 20th century [4,5,6,7,30].

## 4. More Knowledge about Wheat Polymorphism Worldwide

**Figure 2:**
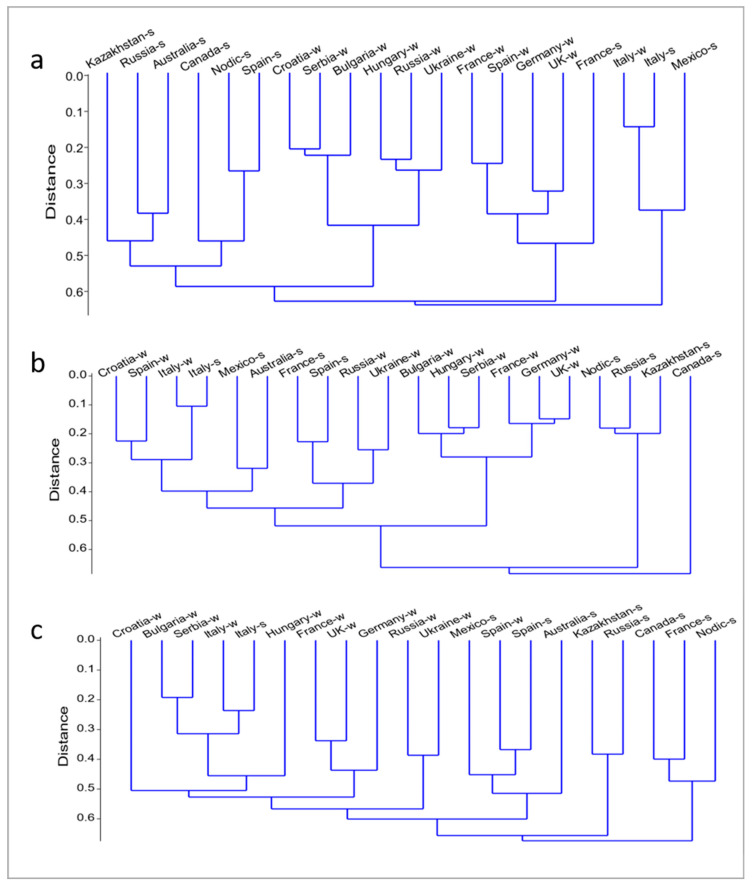
Relationships among genotypes of cultivars bred in different countries and studied for alleles at the *Gli-A1* (**a**), *Gli-D1* (**b**) or *Gli-A2* (**c**) loci. The calculations were performed using the program https://folk.uio.no/ohammer/past/ (accessed on 14 July 2024). “Nordic” is a sum of cultivars studied that were bred in Finland, Norway and Sweden. The list of the origin of the germplasm studied can be provided upon request.

The use of gliadin alleles for genotype identification produced the unexpected discovery that gliadin polymorphism of spring wheat germplasm is highly structured: a group of cultivars bred in a given country has its own characteristic set of *Gli* alleles [31]. In this work, we estimated genetic distances between groups of cultivars bred in different countries worldwide using frequencies of gliadin alleles at each of the six major *Gli* loci separately (Figure 2a–c).

There were some pairs of closely related groups of cultivars, considering any *Gli* locus. For example, Italy spring and Italy winter, Russia winter and Ukraine, Russia spring and Kazakhstan, Germany and the UK. However, for most of the groups of cultivars studied, genotypes of some two groups might be similar for one *Gli* locus differing considerably for other loci. For example, the two groups of Spanish cultivars are close at the *Gli-A2* locus, but not at the *Gli-A1* or *Gli-D1*; two groups of French cultivars differed at each *Gli* locus; cultivars bred in Croatia and Serbia were similar only at the *Gli-A1* locus; and Canada and Mexico at the *Gli-B1* locus, differing strongly at any one of the five other major *Gli* loci (Figure 2 and Dr. Melnik personal communication). Australian and Mexican cultivars were similar at the *Gli-A1* and *Gli-D1*, but strongly differ at each of the other *Gli* loci studied (Figure 2 and Dr. Melnik personal communication), and so on.

Distinct relationships among groups of wheat cultivars studied for different *Gli* loci firmly refute the possibility that the worldwide use of a limited number of “the best” genotypes would reduce the genetic diversity of wheat germplasm as a whole. Moreover, due to the differentiation worldwide of the gliadin polymorphism, the analysis of a few seeds (even one seed) is enough to provide an indication of the origin (country, region) of the grain sample under study.

At the *Gli-A2*, but not at any other *Gli* locus, genotypes of all cultivars studied were divided (except the Italian and Spanish cultivars) into two clear groups, spring and winter. There were two exceptions, the Italy spring group, which was genetically similar to the Italy winter, and other winter groups, and the Spain winter, which occurred between spring groups. Long branches of a tree, especially in the “spring zone” indicated a profound differentiation of the polymorphism at this locus between groups of cultivars bred in different countries (Figure 2c).

## 5. Why Is Wheat Germplasm So Structured?

The analysis of morphological characters, enzymes, grain storage proteins and various DNA markers demonstrated a substantial difference in the frequency of allelic variants and genotypes between wild populations [32,33,34], local varieties and landraces [4,5,6,7,35] of different wheat species. It was suggested that environmental factors (climate, peculiarities of soil, plant diseases) might cause an observed difference in wheat between genotypes [32,34].

There seems to be a logical analogy between a group of cultivars (genotypes) bred in a given region and a group of plants (genotypes) composing a population of wild wheat. Indeed, considerable temporal changes in the assemblage of genotypes caused by natural selection in a particular environment were documented in studies of the experimental populations of common wheat [36,37].

Breeders’ selections for end-use wheat quality, more or less similar in different countries, would not produce this observed worldwide structuring of wheat polymorphism. Thus, we suggest that each newly bred cultivar is always the fruit of the joint efforts of a breeder pursuing breeding aims and of natural selection acting in a given region at all stages of the breeding process, from producing crosses to propagation of the finally selected genotype.

The differentiation of polymorphism of common wheat studied among countries and regions may have adaptive significance, as is proved for wild wheat. Natural selection (caused by eco-climatic conditions) may act in different ways in different regions, limiting the level of genetic diversity of wheat grown in that region: it is known that only a short list of genotypes successful for a given area or region is recommended [38,39]. Therefore, local varieties and landraces may have a particular value in breeding as a source of alleles and their associations improving adaptation of wheat cultivars for the conditions of growth in a given region [40,41,42]. A regionally specific natural selection may cause some kind of “specialization” of polymorphism for each region, thereby increasing differences between genotypes bred in each of these different regions. It has been shown that the genetic diversity of bread wheat at fine spatial and temporal scales depends upon the agricultural region of a country [43]. A great variation in common wheat germplasm (extensive structural rearrangements of DNA sequences and gene content) was assumed to be a result of the history of wheat breeding aimed at improving different wheat characteristics, such as grain yield and quality, resistance to stresses and diseases, and adaptation to conditions of growth [44].

It seems probable that a high level of diversity of common wheat germplasm of the 20th century was, is, and will be maintained over time due to diversity, on a worldwide scale, of natural selection in the different eco-climatic environments of wheat cultivation. Successful genotypes may perhaps contribute to a narrowing of polymorphism at the regional level. However, it is difficult to imagine a wheat genotype that would fit any combination of eco-climatic conditions of growing at the global level of wheat germplasm, or even at a country level. An erosion of genetic diversity of cultivated *T. aestivum* at a species level would be unavoidable only if eco-climatic conditions became identical everywhere wheat is grown.

## 6. Genetic Uniformity Assured? Not for Over 40% of Registrations

The validity of varietal identification for specified quality grades and even forensic analysis is based on the assumption that any one of the varieties involved is distinct, uniform and stable (DUS) with respect to its genotype, including gliadin allelic composition. Based on that, some modern SNP approaches are based on analysis of the DNA of a single plant, which may have been assumed to represent a whole cultivar or accession [26]. However, such an assumption can readily be demonstrated to be erroneous, ignoring the likelihood of diversity in the progeny from a breeding exercise.

**Figure 3:**
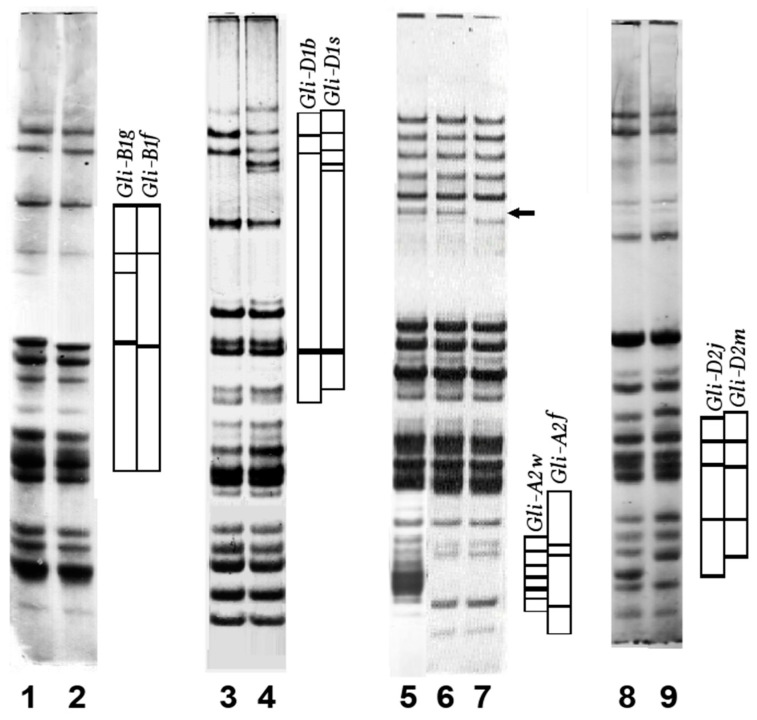
Electropherograms (acid gel) of gliadin of some registered non-uniform common wheat cultivars. Lanes 1, 2, Mercia (UK, year of registration 1984); 3, 4, HY-20 (Canada, 1985); 5, 6, 7, Bezenchukskaya-98 (former USSR, 1951); 8, 9, Suneca (Australia, 1982). Blocks of jointly inherited gliadin bands are shown schematically. The arrow indicates the electrophoretic band encoded by the locus *Gli-B3*.

**Figure 4:**
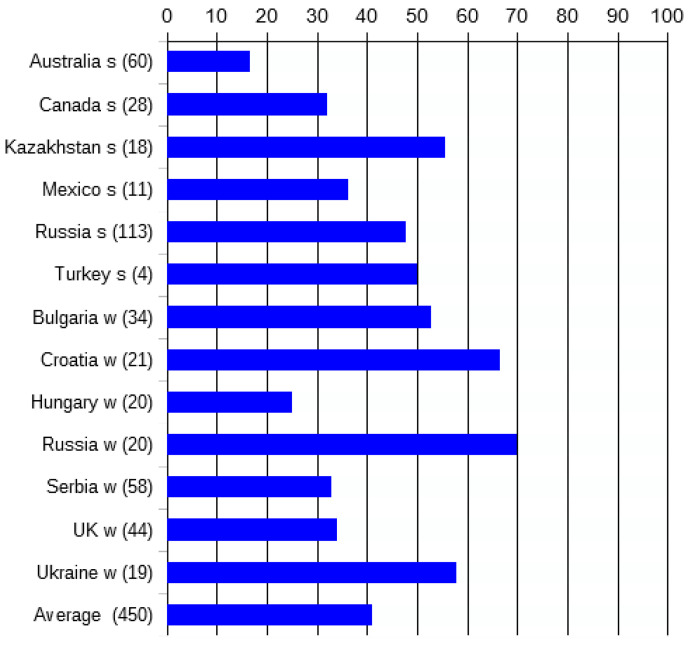
The proportions of non-uniform registered wheat cultivars in major wheat-producing countries show groups having a spring (s) or winter (w) habit. The numbers of cultivars studied for each country are in brackets.

Moreover, seed-by-seed analysis of the grain sample of a cultivar provided evidence of this diversity at the seed level: we documented genetic non-uniformity in many registered common wheat cultivars, in complete transgression of the PBR regulations of DUS (with respect to “Uniformity”) (Figure 3). In total, 450 common wheat cultivars worldwide were studied (at least 8–10 single seeds per cultivar), and many of them (17–70%, from different countries, Figure 4) were found to be composed of multiple authentic biotypes (“authentic” being sister lines originating from the initial cross).

An intra-varietal difference at one *Gli* locus resulted in the presence of two biotypes, at two *Gli* loci—of four biotypes, at three loci—of eight biotypes, and so on. Hence, all the biotypes of a given cultivar appeared to be products of segregation of corresponding heterozygous genotypes. It was confirmed that a non-uniform cultivar inherited all alleles present in its biotypes from parents mentioned in its pedigree. For example, the Australian cultivar “Suneca” is composed of two authentic biotypes differing at the *Gli-D2* locus (Figure 3, lanes 8, 9). One biotype, carrying the allele *Gli-D2m* in its genotype, has obtained it from the parental cultivar “Spica”, while another biotype (with the *Gli-D2j*) obtained it from another parent, “Ciano-67”. In another example, the Italian cultivar “Brasilia”, originated from a complex three-step cross, was found to be composed of 16 authentic biotypes differing at four *Gli* loci (Table 2).

A long time ago, some cases of intra-varietal non-uniformity of common wheat cultivars were described; it was discovered, using seed-by-seed analysis, that individual seeds of a given grain sample might differ in their gliadin genotypes. The different seeds of the same cultivar were considered “biotypes”, especially if the off-type was morphologically identical to the main type of the cultivar [45,46,47].

More recently, knowledge about gliadin genetics has made it possible to distinguish authentic biotypes of a cultivar (sister lines originated from the initial cross) from admixtures of alien genotypes present in grain samples and to establish that cases of non-uniformity are not rare, strange or occasional [48]. From an opposite viewpoint, the existence of authentic biotypes was proven to be general, usual and frequent among registered common wheat cultivars (Figure 4).

Moreover, the quota of non-uniform cultivars registered throughout the 20th century did not change in groups of cultivars bred in Canada (33.3%, and 30.8% among cultivars bred in 1907–74 and 1979–88, respectively), Bulgaria, Croatia, Kazakhstan, Serbia and Omsk (Siberian breeding center of the former USSR), but decreased with time in Australia (25.0%, and 9.4% in 1901–49, and 1966–85, respectively), and in the UK (72.7%, and 18.2% in 1916–77, and 1986–94). There were increases in the Saratov (Volga region) breeding center of the former USSR (42.9%, and 61.5% in 1923–51, and 1972–87, respectively). Therefore, in general, there was no tendency for a decrease, during the 20th century, in the quota of non-uniformity among worldwide newly bred cultivars.

## 7. Is Genetic Uniformity Wholly Desirable?

Many cultivars uniform in gliadin composition could be non-uniform based on analyses for other genetic markers. For example, comparison of the published results of analyses of genotypes of Bulgarian cultivars using microsatellite DNA markers [49] and gliadin polymorphism [25] showed that ten of twenty-nine cultivars, identical in the two studies, were non-uniform in microsatellite analysis and uniform in gliadin alleles, whereas four of the cultivars, uniform in microsatellites, were non-uniform in gliadin composition.

A cultivar that was found to be non-uniform, for example, at one *Gli* locus, may be composed, in fact, of a complex mixture of several (or many) biotypes hidden under the mask of morphological uniformity. The presence of related but different genotypes adds some polymorphism to a given cultivar and thereby may increase its adaptivity and plasticity. Indeed, the relative frequency of biotypes inside a non-uniform cultivar did not change with time when this cultivar grew in the place of its origin but might change drastically when grown in different ecological conditions [48].

Biotypes of a non-uniform cultivar may relate to important wheat characteristics. For example, biotypes of some Australian non-uniform cultivars differed by alleles at the *Glu* loci and by dough quality [50,51]. Moreover, it was proven that changes in the frequency of biotypes in different conditions of growth may influence the grain quality of a given non-uniform cultivar as a whole [48].

## 8. Lessons to Be Learned

The value of wheat genotype identification was demonstrated when analysis of gliadin helped to confirm the theft of a large amount of wheat grain from a storage silo in northern NSW (Australia) [52].

In other work, it was established that the South Ukrainian wheat germplasm can be readily distinguished from any other, in particular, from a Russian one in case of alleged theft. Ukrainian common wheat grain can be easily detected due to two specific genetic markers that are easily distinguished by APAGE, the Gli-A2f and Gli-B2o. The high level of presence (>15–20%), in any grain mixture of seeds with this allelic combination would indicate the presence of the Ukrainian wheat germplasm, and the occurrence of such seeds with a frequency of more than 55% in any batch of grain provides convincing evidence that this batch of grain originates from South-East Ukraine, and nowhere else [53]. The phenomenon of the lack of genetic uniformity of wheat cultivars has not received enough attention recently. No longer can a registered cultivar be called genetically “uniform” only because it is a “registered cultivar”. The presence in a sample of a wheat cultivar of any intra-varietal non-uniformity (biotypes or even a small level of accidental admixture) may lead to the wrong interpretation of results for a scientific experiment. For example, a sound case of damage caused by admixtures to the experimental work occurred in studies of somaclonal variation in common wheat. Our analysis of published gliadin electropherograms of in vitro-produced genotypes strongly differed from the initial ones; they were therefore claimed to be somaclonal variations, but these outlier genotypes were, in fact, either descendants of an occasional out-crossing of the initial genotypes, or admixtures present in the grain samples used in the experiment [54].

Given that agricultural crops, in general, have similar methodologies for crossing and selection, it is likely that the non-uniformity of registered cultivars is prevalent throughout the wide range of agricultural crops. The presence of multiple biotypes goes against the usual requirement of genetic uniformity required for cultivar registration. However, this ruling may impose an unintended penalty on breeders, thereby requiring yet a further hurdle to their progress via selection to registration. Furthermore, it is possible that the presence of multiple biotypes, even as sister lines, offers a minor degree of intra-varietal diversity that provides distinct agronomic advantages. There may thus be a need to re-evaluate relevant rules of cultivar registration for crop species in general.

## Figures and Tables

**Table 1:** The genetic diversity (H = 1 − Σp^2^; ×10^3^) at each major Gli locus for groups of cultivars differing in the year of their registration.

Years	N^1^	*Gli-A1*	*Gli-B1*	*Gli-D1*	*Gli-A2*	*Gli-B2*	*Gli-D2*	Average
1901–1933	46	858	888	764	924	870	867	862 ± 43
1934–1950	60	857	874	695	925	915	881	858 ± 67
1951–1966	102	889	862	818	917	907	911	884 ± 30
1967–1976	183	870	824	787	895	887	895	860 ± 35
1977–1982	191	878	823	694	904	892	877	845 ± 63
1983–1986	143	867	826	662	888	906	873	837 ± 72
1987–1998	143	858	773	695	897	886	842	825 ± 62

Note: N^1^, number of cultivars studied in the group.

**Table 2:** Inheritance of alleles at the *Gli* loci by the cultivars Suneca (Australia, 1982) and Brasilia (Italy, 1985) from its parental cultivars.

Cultivar	*Gli-A1*	*Gli-B1*	*Gli-D1*	*Gli-A2*	*Gli-B2*	*Gli-D2*
**Suneca** **^1^**	*o*	*d*	*f*	*m*	*c*	*m* + *j* **^2^**
Ciano-67	*o*	*d*	*f*	*f*	*c*	*j*
Spica	*o*	*b*	*a*	*m*	*c* + *o*	*m*
**Brasilia**	*a*	*e* + *g*	*a*	*g* + *e*	*v* + *j*	*r* + *a*
Osječka-20	*a*	*g*	*b*	*g*	*j*	*r*
Zlatna-Dolina	*b*	*e*	*a*	*e*	*e*	*m*
Libellula	*a*	*k*	*b*	*g*	*v*	*a*
Bezostaya-1	*b*	*b*	*b*	*b*	*b*	*b*

Notes: **^1^** Non-uniform cultivars are in **bold**; cultivars from their pedigree are listed below. **^2^** The symbol “+” designates the presence of two biotypes differing by alleles at a given *Gli* locus.

## Data Availability

No new data were created or analyzed in this study. Data sharing is not applicable to this article.
